# The association between interpregnancy interval and birth weight: what is the role of maternal polyunsaturated fatty acid status?

**DOI:** 10.1186/1471-2393-13-23

**Published:** 2013-01-25

**Authors:** Luc JM Smits, Hester M Elzenga, Reinoud JBJ Gemke, Gerard Hornstra, Manon van Eijsden

**Affiliations:** 1 Maastricht University Medical Centre, Maastricht, the Netherlands; 2 VU Medical Centre, Amsterdam, the Netherlands; 3 NutriSearch, Maastricht, the Netherlands; 4 Amsterdam Public Health Service (GGD), Amsterdam, the Netherlands

## Abstract

**Background:**

The objective of this study was to evaluate the mediating role of maternal early pregnancy plasma levels of long chain polyunsaturated fatty acids (LCPUFAs) in the association of interpregnancy interval (IPI) with birth weight and smallness for gestational age (SGA) at birth.

**Methods:**

We analysed a subsample of the Amsterdam Born Children and their Development (ABCD) cohort, comprising 1,659 parous pregnant women recruited between January 2003 and March 2004. We used linear and logistic regression to evaluate the associations between fatty acid status, interpregnancy interval and pregnancy outcome.

**Results:**

Low plasma phospholipids concentrations of eicosapentaenoic acid (EPA), docosahexaenoic acid (DHA) and dihomo-gamma-linolenic acid (DGLA), and high concentrations of arachidonic acid (AA) during early pregnancy were associated with reduced birth weight and/or an increased risk of SGA. Short IPIs (< 6 months, with 18–23 months as a reference) were associated with a mean decrease of 207.6 g (SE: ± 73.1) in birth weight (*p* = 0.005) and a twofold increased risk of SGA (OR: 2.05; CI: 0.93–4.51; *p* = 0.074). Adjustment for maternal fatty acid concentrations did not affect these results to any meaningful extent.

**Conclusions:**

Despite the observed association of maternal early pregnancy LCPUFA status with birth weight and SGA, our study provides no evidence for the existence of an important role of maternal EPA, DHA, DGLA or AA in the association of short interpregnancy intervals with birth weight and SGA.

## Background

Interpregnancy intervals (IPIs) shorter than six months are linked with increased risks of preterm birth, lower birth weight and smallness for gestational age (SGA) [1-3]. These adverse pregnancy outcomes are associated with perinatal and neonatal morbidity and mortality and can affect later development and health [4]. Long-term effects have also been described, including increased risks of schizophrenia, menstrual disorders and subfecundity [5-7].

The mechanisms underlying the unfavourable effects of short IPIs on pregnancy outcomes are not well understood. Some causal hypotheses have been put forward, including postpartum hormonal imbalance, maternal stress and the maternal nutritional depletion hypothesis [8-10]. The latter hypothesis states that women with closely spaced births have insufficient time to restore the nutritional reserves needed to support fetal growth in the subsequent pregnancy, resulting in an increased risk of unfavourable pregnancy outcome. Nutrients of interest in the nutritional depletion hypothesis have to meet several criteria: (1) they have to be necessary in pregnancy and fetal development, with shortage leading to an adverse pregnancy outcome; (2) their maternal functional status should decline during pregnancy; and (3) their normalisation to a sufficient concentration after pregnancy has to be slow. The micronutrients folate and iron fit this profile [11,12]. Indeed, in a recent study, the association between short interpregnancy interval and pregnancy outcome was found to be stronger among women with low periconceptional folic acid supplement intake [13].

Nutrients that could play a similar role in the relationship between short interpregnancy interval and adverse pregnancy outcome are the longer-chain, more unsaturated derivatives of the essential fatty acids α-linolenic acid and linoleic acid, also known as long chain polyunsaturated fatty acids (LCPUFAs). This particularly holds for docosahexaenoic acid (DHA, 22:6n-3), eicosapentaenoic acid (EPA, 20:5n-3), arachidonic acid (AA, 20:4n-6) and its precursor, dihomo-gamma-linolenic acid (DGLA, 20:3n-6), which are structural components of cell membranes and are involved in a range of physiologic processes relevant to fetal growth, development and immune system [14-17]. Consequently, a shortage of any of these essential fatty acids could result in a less favourable pregnancy outcome.

A decrease in biochemical LCPUFA status is common in pregnant women [18,19]. This is probably explained by fetal needs during development, since the developing fetus completely depends on the maternal essential fatty acids supply [20]. Complete recovery after delivery takes more than six months [18,21]. A lower absolute (mg/L plasma) and relative (% of total fatty acids) amount of DHA in maternal plasma phospholipids during pregnancy was seen in multigravids compared to primigravids [22], suggesting incomplete recovery of maternal DHA availability after delivery [23]. Low concentrations of most n-3 fatty acids, including DHA, and high concentrations of AA are associated with lower birth weight and/or higher SGA risk [24,25].

In the present study, we evaluated the potential role of the concentrations of DHA, AA, DGLA and EPA in the relationship between interpregnancy interval and adverse pregnancy outcome in a population-based cohort of 1,659 multiparous pregnant women.

## Methods

### Population and design

Data for this study were obtained from the Amsterdam Born Children and their Development (ABCD) study. This prospective, population-based cohort study examined the relationship between maternal lifestyle, ethnicity, psychosocial conditions and nutritional status during pregnancy and the child’s health at birth and in later life (http://www.abcd-study.nl). The study design has been described in detail elsewhere [24]. In short, between January 2003 and March 2004, pregnant women living in Amsterdam were invited to enrol in the study during their first antenatal visit to their obstetric care provider. Detailed information about the mother's socio-demographic data, obstetric history, lifestyle, dietary habits and psychosocial factors was collected by the use of questionnaires, which were available in multiple languages. As part of a biomarker study, blood was drawn after the first prenatal visit to assess the level of fatty acids.

**Figure 1:**
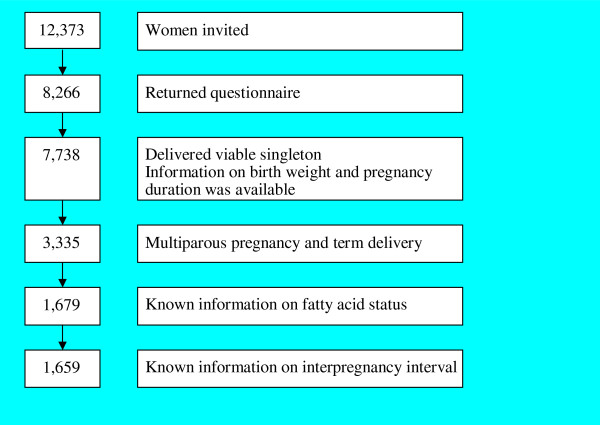
Selection of the study sample.

Of the 12,373 pregnant women invited to participate, 8,266 returned the questionnaire (response rate, 67%). Of this group, 7,738 women gave birth to a single live infant about whom information on birth weight and pregnancy duration was available. For the present study, we excluded all primiparous women (n = 3,993) and women who delivered preterm (delivery before 37 complete weeks of gestation, n = 410). In addition, we excluded 1,656 women (49.9%) who did not participate in the biomarker study and therefore lacked data on fatty acid status, as well as 20 women for whom the interpregnancy interval was not known. Consequently, data about 1,659 women and their infants were available for analysis (see Figure 1).

Study approval was obtained from the Central Committee on Research Involving Human Subjects in the Netherlands, the medical ethical committees of participating hospitals and the Registration Committee of Amsterdam. Written informed consent was obtained from all participants.

### Main variables

Primary outcome variables were birth weight (in grams) and SGA, which was defined as birth weight below the 10th percentile for gestational age based on sex- and parity -specific standards from the Dutch Perinatal Registry (available from the authors upon request). Gestational age at birth was determined by ultrasound (i.e. fetal biometry) or, if unavailable (< 10%), based on the first day of the last menstrual period.

Interpregnancy interval was computed as the amount of time between current and previous delivery dates, diminished by the duration of the current pregnancy. We divided interpregnancy interval into five categories; < 6, 6–11, 12–17, 18–23 and ≥ 24 completed months, and used 18–23 months as the reference category [1,3].

Infant sex, birth weight and gestational age at birth (in weeks) were obtained from the Youth Health Department of the Public Health Service in Amsterdam. The date of the previous delivery was collected via questionnaire.

Fatty acid analysis in the ABCD study was previously described [24]. In short, phospholipids isolated from plasma were saponified and the resulting fatty acids were methylated and measured by capillary gas chromatography with flame ionisation detection. The absolute amounts of arachidonic acid (20:4 n-6), docosahexaenoic acid (22:6 n-3) dihomo-gamma-linolenic acid (20:3 n-6) and eicosapentaenoic acid (20:5 n-3) (in mg/L plasma) were quantified on the basis of the recovery of an internal standard and expressed in a relative value (percentage of total amount of phospholipids-associated fatty acids).

### Potential confounders

A number of variables were considered and treated as potential confounders. Maternal age in years [26] (< 25, 25–34 or ≥ 35), parity [27] (1, 2 or ≥ 3), height [27] (in cm) and pre-pregnancy BMI [27] (in kg/m^2^) were obtained from the questionnaire. A random imputation procedure using linear regression analysis on the available variables was used to complete missing data on height (3.4% missing) and weight (9.5% missing) [24].

Maternal pregnancy-related variables were pregnancy intention (by inquiring whether participants did or did not want to become pregnant) [28], spontaneous pregnancy (by inquiring whether participants became pregnant with or without any medical intervention) [29], time to pregnancy (time needed to become pregnant; < 3, 3–12 or ≥ 12 months) [30] and start of prenatal care in weeks of gestation as calculated from the date of the first prenatal visit, the date of birth and gestational age at birth (<18, 18–23 or ≥24).

Lifestyle variables included self-reported smoking habits [31] and alcohol consumption [32,33] (yes/no) before and/or during pregnancy and psychosocial stress (presence of 0, 1 or ≥ 2 stressors) [34]. Stressors were measured by validated Dutch versions of internationally used questionnaires on depression [35], general anxiety [36], pregnancy-related anxiety [37], parenting stress [38] and work stress [39]. Because there are no internationally accepted thresholds for non-normal scores in pregnant women, we set them at the 90th percentile.

Finally, the selected socio-demographic factors were cohabitant status (living with a partner or living alone) [40], years of education after primary school (< 6, 6–10 or ≥ 11 years) [41] and ethnicity. Ethnicity was determined by asking about the respondent’s country of birth and that of her mother (the Netherlands, Surinam, Turkey, Morocco or other) [42]. All the variables were measured by questionnaire, unless mentioned otherwise.

### Statistical analyses

We examined the relationship between maternal LCPUFA concentrations and pregnancy outcome by means of logistic regression (SGA) and linear regression (birth weight). Because we expected potential associations in the extreme values [24], we used quintiles to categorise the maternal plasma phospholipids concentration of each LCPUFA of interest. The middle category was used as a reference. To examine the relationship between IPI and maternal LCPUFA concentrations, we designated the quintile with the strongest association with adverse pregnancy outcome as the least favourable quintile. We then performed a logistic regression to evaluate whether IPI was associated with being in the least favourable LCPUFA quintile (versus any other LCPUFA quintile).

To evaluate the association between IPI and pregnancy outcome, we performed a logistic regression analysis (SGA) and a linear regression analysis (birth weight). Confounding factors were evaluated by performing multivariate, adjusted analyses in which each of the above-mentioned covariates was separately added to the model. In the SGA analyses, infant sex and gestational age were omitted from the multivariate analyses since they were already part of the definition of SGA. In the logistic regression analyses, a covariate was considered to be a confounder when the odds ratio of the univariate analyses deviated more than 10% after adding that specific covariate. In the linear regression analyses, we regarded a covariate to be a confounder when the deviation of the regression coefficient was greater than 10%. Using this confounder selection strategy, none of the aforementioned factors were identified as confounders, and therefore the final multivariate model only contained interpregnancy interval as an independent variable.

Finally, we assessed the potential influence of maternal LCPUFA concentrations on the relationship between interpregnancy interval and pregnancy outcome by repeating the adjusted logistic and linear regression analyses. In these analyses, LCPUFAs were treated as independent variables, first each LCPUFA separately and then all LCPUFAs simultaneously. Associations were considered statistically significant at p < 0.05. All analyses were conducted with the use of SPSS software (version 15.0; SPSS Inc., Chicago, IL).

## Results

Characteristics of the woman-infant pairs (n = 1659) are presented in Table 1. The mean (± SD) infant birth weight was 3,594 g (± 505), and the mean gestational age at birth was 40.0 ± 1.2 weeks. In total, 12.4% of the infants were born SGA. Of all the included women, 3.6% (n = 60) became pregnant within six months after their previous delivery. Almost half of the women (49.4%; n = 820) had an IPI of more than 24 months, and 13.5% (n = 223) became pregnant again between 18 and 23 months.

**Table 1:** Maternal and infant characteristics (n=1659)

**Characteristic**	
***Infant***	
**Male (%)**	48.1
**Birth weight in g (mean ± SD)**	3594 ± 505
**Gestational age at birth in wk (mean ± SD)**	40.0 ± 1,2
**Small-for-gestational-age (%)**	12.4
***Maternal***	
**Interpregnancy interval (%)**	
< 6 mo	3.6
6-11 mo	16.7
12-17 mo	16.8
18-23 mo	13.5
≥ 24 mo	49.4
**Age (%)**	
< 25 y	5.7
25-34 y	61.2
≥ 35 y	33.1
**Parity (%)**	
1	74.2
2	18.9
≥3	6.9
**Height in cm (mean ± SD)**	168.5 ± 7.2
**Prepregnancy BMI in kg/m**^**2 **^**(mean ± SD)**	23.4 ± 4.1
**Unintended pregnancy (%)**	7.2
**Non-spontaneous pregnancy (%)**	1.9
**Time to pregnancy (%)**	
<3 mo	64.9
3-12 mo	26.4
≥ 12 mo	8.7
**Started prenatal care (%)**	
< 18 wk	92.1
18-23 wk	6.0
≥ 24 wk	1.9
**Alcohol consumption (%)**	
None	41.2
Yes, but not since pregnancy	32.5
Yes, also during pregnancy	26.3
**Smoking (%)**	
None	81.3
Yes, but not since pregnancy	10.2
Yes, also during pregnancy	8.6
**Psychosocial stressors (%)**	
0	73.4
1	16.5
≥2	10.1
**Cohabitant status (% living alone)**	9.2
**Education (%)**	
<6 y	22.3
6-10 y	38.9
≥ 11 y	38.8
**Country of birth (%)**	
Netherlands	64.0
Surinam	6.1
Turkey	4.5
Morocco	7.5
Other non-Western country	11.6
Other Western country	6.0
**Country of birth of the mother’s mother (%)**	
Netherlands	58.4
Surinam	8.4
Turkey	5.1
Morocco	8.3
Other	19.7

### Maternal essential fatty acids and pregnancy outcome

Blood samples for fatty acid analyses were taken at an average gestational age of 13.8 (± 3.5) weeks. Characteristics of the woman-infant pairs who did not take part in the biomarker study (n = 1,656, data not shown) were comparable to those of the women and infants who did. The distributions of quintiles of maternal fatty acid concentrations in plasma phospholipids are listed in Table 2. Compared to infants born to women with intermediate concentrations, infants born to women with concentrations classified in the lowest quintile of EPA, DHA or DGLA weighed 118–183 g less at birth (Table 3). These infants were also more likely to be born SGA, although this association was not statistically significant for DHA.

**Table 2:** **Concentration of the selected fatty acids by quintile in maternal plasma phospholipids at early pregnancy**^**1**^

**Fatty acid**	**Value**	**Quintile**				
	**Mean ± SD**	**Q1**	**Q2**	**Q3**	**Q4**	**Q5**
**Concentration total fatty acid (mg/L)**	1451.81± 246.55	<1246.71	1246.71-1383.61	1383.61-1500.71	1500.71-1639.51	≥1639.51
**Concentration specific fatty acid**^**2**^						
EPA (20:5 n-3)	0.63 ± 0.45	< 0.33	0.33-0.46	0.46- 0.58	0.58 -0.81	≥ 0.81
DHA (22:6 n-3)	4.07 ± 1.12	<3.74	3.74-4.35	4.35-4.86	4.88-5.54	≥ 5.54
DGLA (20:3 n-6)	3.45 ± 0.75	< 2.83	2.83-3.23	3.23- 3.56	3.56- 4.05	≥ 4.05
AA (20:4 n-6)	9.48 ± 1.68	< 8.06	8.06- 8.99	8.99- 9.83	9.83-10.86	≥ 10.86

^1 ^n=1659; Blood assessment in early pregnancy at an average pregnancy duration of 13.5 ±3.3 weeks of gestation. EPA, eicosapentaenoic acid (20:5 n-3); DHA, docosahexaenoic acid (22:6 n-3), DGLA; dihomo-γ-linolenic acid (20:3 n-6), AA; arachidonic acid (20:4 n-6).

**Table 3:** **Results of the regression analyses relating essential fatty acid concentrations in quintiles and pregnancy outcome, defined as small for gestational age SGA and birth weight**^**1**^

**Fatty acid**	**Birth Weight (g)**		**SGA**	
	**Mean ± SD**	**Beta ± SE**^**2**^	**% of women with SGA birth within quintile**	**Odds ratio (95% CI)**^**3**^
**EPA**				
Q1	3595 ± 536	−182.5 ± 39.0^4^	12.7	2.09 (1.32-3.30) ^4^
Q2	3579 ± 542	−66.1 ± 39.0	13.3	1.42 (0.88-2.31)
Q3	3645 ± 474	0.0	9.7	1.00
Q4	3664 ± 498	18.4 ± 38.9	9.6	0.98 (0.59-1.64)
Q5	3619 ± 489	−26.6 ± 39.0	10.8	1.13 (0.68-1.87)
**DHA**				
Q1	3518 ± 520	−118.2 ± 39.2^4^	13.3	1.11 (0.70-1.75)
Q2	3593 ± 539	−43.8 ± 39.2	12.7	1.05 (0.66-1.67)
Q3	3636 ± 505	0.0	12.1	1.00
Q4	3602 ± 471	−34.4 ± 39.2	11.7	0.96 (0.60-1.54)
Q5	3621 ± 484	−15.4 ± 39.2	12.0	0.99 (0.62-1.59)
**DGLA**				
Q1	3467 ± 472	−127.1 ± 38.9^4^	17.2	1.72 (1.10-2.69)^4^
Q2	3610 ± 520	16.5 ± 38.9	14.8	1.43 (0.90-2.26)
Q3	3594 ± 531	0.0	10.8	1.00
Q4	3634 ± 495	40.0 ± 38.9	11.5	1.07 (0.66-1.74)
Q5	3665 ± 486	71.0 ± 38.9	7.5	0.67 (0.39-1.15)
**AA**				
Q1	3606 ± 507	−52.8 ± 39.1	10.8	1.07 (0.65-1.76)
Q2	3617 ± 501	−42.2 ± 39.1	9.7	0.94 (0.57-1.57)
Q3	3659 ± 482	0.0	10.2	1.00
Q4	3574 ± 533	−85.0 ± 39.0^4^	13.5	1.37 (0.86-2.21)
Q5	3514 ± 494	−145.0 ± 39.1^4^	17.6	1.88 (1.19-2.95)^4^

^1 ^Linear regression analysis with birth weight as dependent variable and fatty acid concentration as independent variable. n=1659 mother-infant pairs. Q1 to Q5 represents the quintile distribution of the relative concentrations given in Table 3. EPA, eicosapentaenoic acid (20:5 n-3); DHA, docosahexaenoic acid (22:6 n-3), DGLA; dihomo-γ-linolenic acid (20:3 n-6), AA; arachidonic acid (20:4 n-6). The middle quintile, Q3, used as reference category.

^2 ^Difference between the mean birth weight in the specific quintile and that of the reference quintile.

^3 ^Odds ratio (95% CI).

^4^*p*<0.05.

A statistically significant association between maternal AA concentrations and birth weight and SGA was also present. Women with AA concentrations in the highest quintile gave birth to infants with birth weights 145.0 g (SE: 39.1) lower and a nearly two times increased chance of SGA in comparison with women with AA concentrations in the middle quintile (OR = 1.88 (95% CI: 1.19–2.95).

### Interpregnancy interval and maternal LCPUFA concentrations

We did not observe any statistically significant associations between short interpregnancy intervals and maternal LCPUFA concentrations (Table 4). Compared to women with an IPI of 18–23 months, mothers with long IPIs (≥ 24 months) had a significantly higher probability of having lower EPA and higher AA concentrations in their plasma phospholipids during the first trimester.

**Table 4:** Results of the logistic regression analysis of the association between interpregnancy interval and unfavorable maternal essential fatty acid status

**Interpregnancy interval (mo)**		**EPA**^**1**^			**DHA**^**1**^			**DGLA**^**1**^			**AA**^**1**^		
	**N**	**% women in 1st Quintile**	**Mean ± SD concentration**	**Odds ratio**^**2 **^**(95% CI)**	**% women in 1st Quintile**	**Mean ± SD concentration**	**Odds ratio**^**2 **^**(95% CI)**	**% women in 1st Quintile**	**Mean ± (SD) concentration**	**Odds ratio**^**2 **^**(95% CI)**	**% women in 5**^**th **^**Quintile**	**Mean (±SD) concentration**	**Odds ratio**^**2 **^**(95% CI)**
< 6	60	23.3	0.66 ± 0.47	1.58 (0.79-3.17)	18.3	4.77 ± 1.20	1.60 (0.51-2.22)	25.0	3.30 ± 0.69	1.22 (0.62-2.37)	25.0	9.34 ± 1.71	1.09 (0.52-2.29)
6-11	276	11.2	0.68 ± 0.44	0.66 (0.39-1.10)	18.3	4.70 ± 1.08	1.12 (0.71-1.77)	22.8	3.41 ± 0.75	1.08 (0.71-1.65)	21.7	9.28 ± 1.56	0.97 (0.52-2.29)
12-17	280	11.8	0.67 ± 0.38	0.69 (0.42-1.16)	19.2	4.85 ± 1.08	0.81 (0.50-1.31	17.9	3.54 ± 0.74	0.79 (0.51-1.23)	21.1	9.28 ± 1.47	0.79 (0.49-1.28)
18-23	223	16.1	0.70 ± 0.53	1 (ref)	14.6	4.75 ± 1.13	1 (ref)	21.5	3.40 ± 0.70	1 (ref)	22.9	9.35 ± 1.72	1 (ref)
≥ 24	820	26.6	0.58 ± 0.45	1.88 (1.28-2.78)	17.3	4.57 ± 1.14	1.40 (0.96-2.06)	18.9	3.46 ± 0.77	0.85 (0.59-1.22)	17.9	9.66 ± 1.76	1.53 (1.04-2.25)
Total	1659		0.63 ± 0.45			4.67 ± 1.12			3.45 ± 0.75			9.48 ± 1.68	

^1 ^EPA, eicosapentaenoic acid (20:5 n-3); DHA, docosahexaenoic acid (22:6 n-3), DGLA; dihomo-γ-linolenic acid (20:3 n-6), AA; arachidonic acid (20:4 n-6).

^2 ^Odds ratios resulting from logistic regression analysis of the association of interpregnancy interval with having an unfavorable blood concentration of either of four essential fatty acids. For each fatty acid, the quintile having strongest association with adverse pregnancy outcome (see Table 3) was designated as the unfavorable fatty acid outcome. (EPA: Quintile 1, DHA: Quintile 1, DGLA: Quintile 1, AA: Quintile 5).

### Interpregnancy interval and pregnancy outcome

Both short (< 6 months) and long (≥ 24 month) IPIs were significantly associated with lower birth weight (Table 5). The short interval category showed an estimated difference in birth weight of −206.7 g (SE: ± 73.1) compared to the reference category (18–23 months). We also observed a corresponding increase in SGA risk, though this was not statistically significant. Women with an IPI of less than six months were twice as likely to deliver an SGA child than women who became pregnant 18–23 months after their previous delivery (OR: 2.05; 95% CI: 0.93, 4.51). None of the selected maternal and infant characteristics acted as confounding factors.

**Table 5:** **Results of regression analyses of interpregnancy interval and pregnancy outcome (birth weight and SGA) adjusted for maternal LCPUFAs concentrations in early pregnancy**^**1**^

**Interpregnancy interval (mo)**		**Birth weight**			**Small for Gestational Age (SGA)**		
		**Unadjusted**		**Adjusted for LCPUFAs**^**2**^	**Unadjusted**		**Adjusted for LCPUFAs**^**2**^
	**n**	**Mean ±SD**	**Beta ± SE**	**Beta ± SE**	**Percentage (%)**	**Odds ratio (95%CI)**	**Odds ratio (95%CI)**
<6	60	3500 ± 489	−206.7 ± 73.1^3^	−193.4 ± 72.1^3^	18.3	2.05 (0.93-4.51)	1.92 (0.86-4.28)
6-11	276	3628 ± 478	−78.5 ± 45.3	−84.1 ± 44.8	9.1	0.91 (0.50-1.66)	0.93 (0.51-1.72)
12-17	280	3613 ± 483	−94.1 ± 45.1^4^	−115.0 ± 44.6	9.3	0.94 (0.52-1.70)	0.93 (0.51-1.72)
18-23	223	3707 ± 499	1.0	1.0	.9.9	1.00	1.00
≥24	820	3552 ± 519	−154.7 ± 38.0^3^	−135.4 ± 37.8^3^	14.8	1.58 (0.98-2.56)	1.43 (0.88-2.33)

^1 ^Linear regression analysis for birth weight as dependent variable and interpregnancy interval in categories as independent variable; logistic regression analysis for SGA as dependent and interpregnancy interval in categories as independent variable. SGA was defined as birth weight < 10^th^ percentile for gestational age based on sex- and parity-specific standards. n=1659 mother-infant pairs. None of the selected maternal and infant characteristics acted as confounding factors.

^2 ^Analysis adjusted for all four selected fatty acids together.

^3 ^Significantly different from reference category (18–23 mo) *p*<0.005.

^4 ^Significantly different from reference category (18–23 mo) *p*<0.05.

### Role of maternal essential fatty acid status in the relationship between interpregnancy interval and pregnancy outcome

After adjusting for each LCPUFA separately, the regression analyses on interpregnancy interval and birth weight/SGA did not show any important changes in regression coefficients or in odds ratios. When the four selected fatty acids were adjusted for simultaneously, there was no meaningful change in the observed relationships either (Table 5). Results were not materially different when LCPUFA variables were entered as quintiles instead of continuous variables (results not displayed).

## Discussion

This study examined the potential role of maternal LCPUFA status during early pregnancy in the association between short interpregnancy intervals with birth weight and SGA. Our results showed an association between IPIs of less than six months (and ≥ 24 months) and adverse pregnancy outcome (Table 5). These findings correspond to earlier observations within the ABCD cohort on the relationship between folate, interpregnancy interval and birth weight, [13] as well as with the findings of a meta-analysis of studies of the effects of short IPIs [1].

Our analyses of the relationship between maternal essential fatty acids and pregnancy outcome (Table 3) showed an increased risk of lower birth weight and/or SGA in women with low EPA, DHA and DGLA plasma phospholipids concentrations during early pregnancy. For AA, on the contrary, higher concentrations were associated with lower mean birth weights and increased risk of SGA. These results correspond with earlier observations in the ABCD cohort including nulliparas [24]. A recent study of 782 women by Dirix et al. [25] described a relationship between increased birth weight and increased DHA concentrations measured in maternal plasma phospholipids in early pregnancy. The authors also reported that birth weight decreased as AA contents increased in late pregnancy. In contrast to our results, their findings on DGLA concentrations were in the same line as AA, with a negative association between maternal concentrations at delivery and birth weight.

Our analyses of interpregnancy interval and maternal LCPUFA concentrations did not reveal any meaningful associations between IPIs and individual LCPUFA concentrations (Table 4). In particular, no significant associations were observed for IPIs shorter that six months which have been shown (here and in other analyses) to be associated with adverse pregnancy outcomes. To our knowledge, the association between short IPI and LCPUFA status during a new pregnancy was not examined before. Findings from a study conducted by Al et al. [18] on maternal essential fatty acid and LCPUFA patterns during and after pregnancy imply a ‘recovery time’ of at least six months for the biochemical DHA status to normalise after pregnancy. Our results, however, indicated that fatty acid status as measured during pregnancy is not different for women with either short or intermediate interpregnancy intervals.

After adjusting for the selected maternal LCPUFA concentrations, we did not observe any meaningful changes in the relationship between interpregnancy interval and birth weight and/or risk of SGA (Table 5). The results of this study therefore do not support the hypothesis that maternal DGLA, AA, EPA or DHA concentrations during early pregnancy play an important role in the relationship between short IPI and adverse pregnancy outcome. This is likely due to the lack of association between short IPIs and maternal LCPUFA concentrations during early pregnancy.

Although 50% of the potential study population was excluded because of a lack of information on their fatty acid concentrations, a comparison of maternal and infant characteristics between included and excluded participants indicated that the resulting study population was representative for all. Because of a potential lack of power, we also decided not to investigate less common outcomes such as preterm birth (n = 64, 3.9%) or infant death. Since this study focused on birth weight and SGA, which are clinically relevant outcome measures, restricting the study to term-born infants made it possible to investigate birth weight as a reflection of health outcomes without the distorting effect of preterm delivery [43].

None of the preselected maternal and infant covariables acted as confounders in the association between IPI and birth weight/SGA. However, we cannot exclude the possibility of residual confounding due to unmeasured confounders or misclassification as a result of recall problems.

Measurement of essential fatty acid and LCPUFA concentrations in early pregnancy reflects the potential shortage due to a short interpregnancy interval. Moreover, this early stage of pregnancy is vitally import for fetal development [17,44,45] and is places a great demand on maternal nutrients. Although much is known about the essence and biological function of essential fatty acids in human life and pregnancy, we are still lacking basic information such as the range of ‘healthy’ concentrations of every fatty acid. Therefore, we chose to use the quintile found to be associated with the highest risk of adverse pregnancy outcome for the analysis of the influence of the selected LCPUFA concentration (quintile) on the relationship between IPI and birth weight and risk of SGA.

## Conclusion

In conclusion, the study results do not support the idea that maternal early pregnancy DHA, AA, EPA or DGLA levels play an important role in the maternal depletion hypothesis. Although maternal supplementation may be beneficial for individual cases, based on the results of the current study we do not expect that routine periconceptional maternal fatty acid supplementation will mitigate the adverse effects of short IPIs on pregnancy outcomes.

## Competing interests

None of the authors declare having financial or non-financial competing interests.

## Authors’ contributions

LS conceived of the study and developed the design of the analyses, HE performed the statistical analyses and drafted the manuscript, MvE participated in the design and data collection from the ABCD study, and all authors read and approved the final manuscript.

## Pre-publication history

The pre-publication history for this paper can be accessed here:

http://www.biomedcentral.com/1471-2393/13/23/prepub

## References

[B1] Conde-Agudelo A Rosas-Bermudez A Kafury-Goeta AC Birth spacing and risk of adverse perinatal outcomes: a meta-analysis JAMA 2006 295 1809 1823 10.1001/jama.295.15.1809 16622143

[B2] Rodrigues T Barros H Short interpregnancy interval and risk of spontaneous preterm delivery Eur J Obstet Gynecol RB 2008 136 184 188 10.1016/j.ejogrb.2007.03.014 17490802

[B3] Zhu BP Effect of interpregnancy interval on birth outcomes: findings from three recent US studies Int J Gynaecol Obstet 2005 89 Suppl 1 S25 S33 15820365 10.1016/j.ijgo.2004.08.002

[B4] Fedrick J Adelstein P Influence of pregnancy spacing on outcome of pregnancy BMJ 1973 4 753 756 10.1136/bmj.4.5895.753 4758571 PMC1588018

[B5] Smits L Pedersen C Mortensen P van Os J Association between short birth intervals and schizophrenia in the offspring Schizophr Res 2004 70 49 56 10.1016/j.schres.2003.10.002 15246463

[B6] Smits L Zielhuis G Jongbloet P Bouchard G The association of birth interval, maternal age and season of birth with the fertility of daughters: a retrospective cohort study based on family reconstitutions from nineteenth and early twentieth century Quebec Paediatr Perinat Epidemiol 1999 13 408 420 10.1046/j.1365-3016.1999.00215.x 10563360

[B7] Smits LJ Willemsen WN Zielhuis GA Jongbloet PH Conditions at conception and risk of menstrual disorders Epidemiology 1997 8 524 529 10.1097/00001648-199709000-00009 9270954

[B8] Winkvist A Rasmussen KM Habicht JP A new definition of maternal depletion syndrome Am J Public Health 1992 82 691 694 10.2105/AJPH.82.5.691 1566948 PMC1694126

[B9] Miller JE Birth intervals and perinatal health: an investigation of three hypotheses Fam Plann Perspect 1991 23 62 70 10.2307/2135451 2060613

[B10] Smits LJ Jongbloet PH Zielhuis GA Preovulatory overripeness of the oocyte as a cause of ovarian dysfunction in the human female Med Hypotheses 1995 45 441 448 10.1016/0306-9877(95)90218-X 8748083

[B11] Smits LJ Essed GG Short interpregnancy intervals and unfavourable pregnancy outcome: role of folate depletion Lancet 2001 358 2074 2077 10.1016/S0140-6736(01)07105-7 11755634

[B12] King JC The risk of maternal nutritional depletion and poor outcomes increases in early or closely spaced pregnancies J Nutr 2003 133 5 Suppl 2 1732S 1736S 12730491 10.1093/jn/133.5.1732S

[B13] Van Eijsden M Smits LJ van der Wal MF Bonsel GJ Association between short interpregnancy intervals and term birth weight: the role of folate depletion Am J Clin Nutr 2008 88 147 153 18614735 10.1093/ajcn/88.1.147

[B14] Hornstra G Essential fatty acids in mothers and their neonates Am J Clin Nutr 2000 71 5 Suppl 1262S 1269S 10799400 10.1093/ajcn/71.5.1262s

[B15] Das UN Essential fatty acids - a review Curr Pharm Biotechnol 2006 7 467 482 10.2174/138920106779116856 17168664

[B16] Calder PC Krauss-Etschmann S de Jong EC Dupont C Frick JS Frokiaer H Early nutrition and immunity - progress and perspectives Br J Nutr 2006 96 774 790 17010239

[B17] Innis SM Fatty acids and early human development Early Hum Dev 2007 83 761 766 10.1016/j.earlhumdev.2007.09.004 17920214

[B18] Al MD van Houwelingen AC Kester AD Hasaart TH de Jong AE Hornstra G Maternal essential fatty acid patterns during normal pregnancy and their relationship to the neonatal essential fatty acid status Br J Nutr 1995 74 55 68 10.1079/BJN19950106 7547829

[B19] Otto SJ Houwelingen AC Antal M Manninen A Godfrey K Lopez-Jaramillo P Maternal and neonatal essential fatty acid status in phospholipids: an international comparative study Eur J Clin Nutr 1997 51 232 242 10.1038/sj.ejcn.1600390 9104573

[B20] Haggarty P Effect of placental function on fatty acid requirements during pregnancy Eur J Clin Nutr 2004 58 1559 1570 10.1038/sj.ejcn.1602016 15266306

[B21] Otto SJ van Houwelingen AC Badart-Smook A Hornstra G Comparison of the peripartum and postpartum phospholipid polyunsaturated fatty acid profiles of lactating and nonlactating women Am J Clin Nutr 2001 73 1074 1079 11382662 10.1093/ajcn/73.6.1074

[B22] Al MD van Houwelingen AC Hornstra G Relation between birth order and the maternal and neonatal docosahexaenoic acid status Eur J Clin Nutr 1997 51 548 553 10.1038/sj.ejcn.1600444 11248881

[B23] Van den Ham EC van Houwelingen AC Hornstra G Evaluation of the relation between n-3 and n-6 fatty acid status and parity in nonpregnant women from the Netherlands Am J Clin Nutr 2001 73 622 627 11237941 10.1093/ajcn/73.3.622

[B24] Van Eijsden M Hornstra G van der Wal MF Vrijkotte TG Bonsel GJ Maternal n-3, n-6, and trans fatty acid profile early in pregnancy and term birth weight: a prospective cohort study Am J Clin Nutr 2008 87 887 895 18400711 10.1093/ajcn/87.4.887

[B25] Dirix CE Kester AD Hornstra G Associations between neonatal birth dimensions and maternal essential and trans fatty acid contents during pregnancy and at delivery Br J Nutr 2009 101 399 407 10.1017/S0007114508006740 18613984

[B26] Newburn-Cook CV Onyskiw JE Is older maternal age a risk factor for preterm birth and fetal growth restriction? a systematic review Health Care Women Int 2005 26 852 875 10.1080/07399330500230912 16214797

[B27] Cogswell ME Yip R The influence of fetal and maternal factors on the distribution of birthweight Semin Perinatol 1995 19 222 240 10.1016/S0146-0005(05)80028-X 7570074

[B28] Mohllajee AP Curtis KM Morrow B Marchbanks PA Pregnancy intention and its relationship to birth and maternal outcomes Obstet Gynecol 2007 109 678 686 10.1097/01.AOG.0000255666.78427.c5 17329520

[B29] Helmerhorst FM Perquin DA Donker D Keirse MJ Perinatal outcome of singletons and twins after assisted conception: a systematic review of controlled studies BMJ 2004 328 261 10.1136/bmj.37957.560278.EE 14742347 PMC324454

[B30] Basso O Baird DD Infertility and preterm delivery, birthweight, and caesarean section: a study within the Danish national birth cohort Hum Reprod 2003 18 2478 2484 10.1093/humrep/deg444 14585905

[B31] Crawford JT Tolosa JE Goldenberg RL Smoking cessation in pregnancy: why, how, and what next Clin Obstet Gynecol 2008 51 419 435 10.1097/GRF.0b013e31816fe9e9 18463471

[B32] Livy DJ Maier SE West JR Long-term alcohol exposure prior to conception results in lower fetal body weights Birth Defects Res B Dev Reprod Toxicol 2004 71 135 141 10.1002/bdrb.20007 15282734

[B33] Kuczkowski KM The effects of drug abuse on pregnancy Curr Opin Obstet Gynecol 2007 19 578 585 10.1097/GCO.0b013e3282f1bf17 18007137

[B34] Hobel CJ Goldstein A Barrett ES Psychosocial stress and pregnancy outcome Clin Obstet Gynecol 2008 51 333 348 10.1097/GRF.0b013e31816f2709 18463464

[B35] Radloff LS The CES-D scale: a self-report depression scale for research in the general population Appl Psychol Meas 1977 1 385 401 10.1177/014662167700100306

[B36] Spielberger CD STAI manual for the state-trait anxiety inventory 1970 Washington: Consulting Psychologists Press

[B37] Huizink AC Mulder EJ Robles de Medina PG Visser GH Buitelaar JK Is pregnancy anxiety a distinctive syndrome? Early Hum Dev 2004 79 81 91 10.1016/j.earlhumdev.2004.04.014 15324989

[B38] Crnic KA Greenberg MT Minor parenting stresses with young children Child Dev 1990 61 1628 1637 10.2307/1130770 2245752

[B39] Karasek R Brisson C Kawakami N Houtman I Bongers P Amick B The Job content questionnaire (JCQ): an instrument for internationally comparative assessments of psychosocial job characteristics J Occup Health Psychol 1998 3 322 355 9805280 10.1037//1076-8998.3.4.322

[B40] Luo ZC Wilkins R Kramer MS Disparities in pregnancy outcomes according to marital and cohabitation status Obstet Gynecol 2004 103 1300 1307 10.1097/01.AOG.0000128070.44805.1f 15172868

[B41] Bibby E Stewart A The epidemiology of preterm birth Neuro Endocrinol Lett 2004 25 Suppl 1 43 47 15735585

[B42] Troe EJ Raat H Jaddoe VW Hofman A Looman CW Moll HA Explaining differences in birthweight between ethnic populations. The generation R study BJOG 2007 114 1557 1565 10.1111/j.1471-0528.2007.01508.x 17903227

[B43] Wilcox AJ On the importance - and the unimportance - of birthweight Int J Epidemiol 2001 30 1233 1241 10.1093/ije/30.6.1233 11821313

[B44] Otto SJ van Houwelingen AC Badart-Smook A Hornstra G Changes in the maternal essential fatty acid profile during early pregnancy and the relation of the profile to diet Am J Clin Nutr 2001 73 302 307 11157328 10.1093/ajcn/73.2.302

[B45] Van Houwelingen AC Puls J Hornstra G Essential fatty acid status during early human development Early Hum Dev 1992 31 97 111 10.1016/0378-3782(92)90038-I 1292926

